# Identification of new candidate therapeutic target genes in head and neck squamous cell carcinomas

**DOI:** 10.18632/oncotarget.10163

**Published:** 2016-06-18

**Authors:** Marie-Paule Sablin, Coraline Dubot, Jerzy Klijanienko, Sophie Vacher, Lamia Ouafi, Walid Chemlali, Martial Caly, Xavier Sastre-Garau, Emmanuelle Lappartient, Odette Mariani, José Rodriguez, Thomas Jouffroy, Angélique Girod, Valentin Calugaru, Caroline Hoffmann, Rosette Lidereau, Frédérique Berger, Maud Kamal, Ivan Bieche, Christophe Le Tourneau

**Affiliations:** ^1^ Department of Medical Oncology, Institut Curie, Paris and Saint-Cloud, France; ^2^ Unit of Pharmacogenomics, Department of Genetics, Institut Curie, Paris, France; ^3^ Department of Biopathology, Institut Curie, Paris, France; ^4^ Department of Surgery, Institut Curie, Paris, France; ^5^ Department of Radiotherapy, Institut Curie, Paris, France; ^6^ Department of Biostatistics, Institut Curie, Paris, France; ^7^ EA7331, Paris Descartes University, Sorbonne Paris Cité, Faculty of Pharmaceutical and Biological Sciences, Paris, France; ^8^ EA7285, Versailles-Saint-Quentin-en-Yvelines University, Versailles, France

**Keywords:** head and neck squamous cell carcinoma, gene expression, clinical prognostic and theranognostic biomarkers

## Abstract

**Background:**

We aimed at identifying druggable molecular alterations at the RNA level from untreated HNSCC patients, and assessing their prognostic significance.

**Methods:**

We retrieved 96 HNSCC patients who underwent primary surgery. Real-time quantitative RT-PCR was used to analyze a panel of 42 genes coding for major druggable proteins. Univariate and multivariate analyses were performed to assess the prognostic significance of overexpressed genes.

**Results:**

Median age was 56 years [35–78]. Most of patients were men (80%) with a history of alcohol (70.4%) and/or tobacco consumption (72.5%). Twelve patients (12%) were HPV-positive. Most significantly overexpressed genes involved cell cycle regulation (CCND1 [27%], CDK6 [21%]), tyrosine kinase receptors (*MET* [18%], *EGFR* [14%]), angiogenesis (*PGF* [301%], *VEGFA* [14%]), and immune system (*PDL1/CD274* [28%]). *PIK3CA* expression was an independent prognostic marker, associated with shorter disease-free survival.

**Conclusions:**

We identified druggable overexpressed genes associated with a poor outcome that might be of interest for personalizing treatment of HNSCC patients.

## INTRODUCTION

Head and neck squamous cell carcinoma is the fifth most common cancer worldwide [1]. Multimodal management of non-metastatic disease includes surgery, radiotherapy, and chemotherapy. Prognosis of patients remains poor when they recur, with more than half of locally advanced HNSCC patients who recur [2, 3].

The Cancer Genome Atlas (TCGA) recently published the whole genome analysis of HNSCC, reporting DNA mutations, gene copy number alterations and main altered expressed genes [4]. Cetuximab, that targets EGFR, is the only approved targeted agent in combination with radiotherapy for HNSCC treatment in the recurrent and/or metastatic setting [5, 6]. Cetuximab lacks a predictive biomarker of efficacy.

We aimed in our study at identifying druggable molecular alterations at the RNA level from untreated HNSCC patients, and at assessing their prognostic significance in order to find new targetable genes that could be of interest for new treatment strategies.

## RESULTS

### Patient characteristics

We analyzed tumor samples from 96 untreated HNSCC patients treated with primary surgery at Institut Curie (Paris, France) between 1990 and 2006. Patient characteristics are presented in Table 1. Median follow-up was 125 months (range: 2.6 days to 236 months). Median age was 56, most of patients were male and heavy smokers (mean pack years = 19). Twelve patients had HPV-positive tumors (Table 1).

**Table 1:** Clinical, biological and pathological characteristics of the 96 HNSCC patients, in relation with disease-free interval (DFI)

	Patients (%)	Events^a^(%)	DFI^b^
*Total*	96 (100)	45 (46.8)	
*Age*			
< 56	46 (47.9)	23 (42.6)	0.62 (NS)
≥ 56	50 (52.1)	22 (52.4)	
*Sexe*			
Female	19 (19.8)	8 (42.1)	0.81 (NS)
Male	77 (80.2)	37 (48.1)	
*Alcool^c^*			
Yes	50 (70.4)	24 (48)	0.32 (NS)
No	21 (29.6)	8 (38.1)	
*Tobacco^d^*			
Yes	58 (72.5)	28 (48.3)	0.42 (NS)
No	22 (27.5)	7 (31.8)	
*HPV*			
Negative	84 (87.5)	42 (50)	**0.036**
Positive	12 (12.5)	3 (25)	
*AJCC stage*			
Stage I	10 (10.4)	5 (50)	0.69 (NS)
Stage II	15 (15.6)	6 (40)	
Stage III	12 (12.5)	4 (33.3)	
Stage IV	59 (61.5)	30 (50.8)	
*Tumor location*			
Oral cavity	43 (44.8)	22 (51.2)	0.072 (NS)
Larynx	17 (17.7)	8 (47.1)	
Oropharynx	20 (20.8)	5 (25)	
Hypopharynx	16 (16.7)	10 (62.5)	

a Events: local or metastatic recurrence, second cancer.

b Log-rank test.

c Tobacco use was considered at 10 packyears or more. Information available for 71 patients.

d Alcohol use was considered at 10 gr/day or more (ie. alcohol unit). Information available for 80 patients.

DFI: disease-free interval; NS: not significant; HNSCC: head and neck squamous cell carcinoma.

AJCC: American Joint Committee on Cancer.

### mRNA expression of the 42 targetable genes

To determine the cut-off point for the 42 genes expression in HNSCCs, all mRNA values were determined for the 27 normal head and neck RNA samples. As these values remained under 3, normalized mRNA values of 3 or more were considered to represent gene overexpression in tumor samples. We previously used the same cut-off points for tumor gene overexpression [7].

The mRNA levels of all targetable genes were detectable and quantifiable by real-time quantitative RT-PCR based on fluorescence SYBR Green method (Cycle Threshold, Ct < 32), in both the normal and tumorous head and neck tissue (except for *ALK* and *ROS1*). Medians and ranges of mRNA levels for the 40 expressed genes and controls *CCND1*, *MKI67* are shown in Table 2, along with the percentages of gene overexpression. In our series of 96 HNSCCs, *PGF, PDL1/CD274, CDK6, MET, EGFR* and *VEGFA* were the most frequently over-expressed genes in respectively 30%, 28%, 21%, 18%, 14% and 14% of the samples. Six additional genes were overexpressed in 3% to 8% of the samples (i.e. *IGF1R, RET, CDK4, KITLG, PDGFRB* and *PIK3CA*). *CCND1* and *MKI67*, used as prognostic controls, were overexpressed in 27% and 31% of samples, respectively.

**Table 2:** mRNA expression of 40 expressed druggable genes and *CCND1, MKI67* in HNSCC relative to normal head and neck tissue and percentages of overexpressed tumors

Genes	Ct median of normal head and neck tissue (*n*= 27)	Normal head and neck tissue(*n*= 27)	HNSCC (*n* = 96)	% of overexpressed tumors (N target > 3)
***PGF***	29.12 (27.97–30.25)^a^	1.00 (0.37–2.26)^b^	1.93 (0.57–15.5)	***30.2%***
***PDL1***	28.60 (26.35–32.59)	1.00 (0.12–2.95)	1.67 (0.06–23.40)	***28.1%***
***CDK6***	25.29 (23.88–27.41)	1.00 (0.32–1.62)	2.25 (0.33–8.32)	***20.8%***
***MET***	27.28 (25.33–28.42)	1.00 (0.30–1.91)	1.87 (0.21–16.5)	***17.7%***
***EGFR***	26.00 (24.08–27.31)	1.00 (040–2.12)	1.51 (0.21–46.1)	***13.5%***
***VEGFA***	25.70 (23.64–28.17)	1.00 (0.34–2.32)	1.43 (0.19–10.25)	***13.5%***
***IGF1R***	27.97 (26.07–30.90)	1.00 (0.29–2.50)	1.36 (0.18–4.62)	8.3%
***RET***	29.86 (26.86–32.75)	1.00 (0.16–2.90)	0.57 (0.05–26.5)	7.3%
***CDK4***	24.95 (24.11–26.67)	1.00 (0.67–1.38)	1.39 (0.76–4.19)	7.3%
***KITLG***	27.76 (21.04–29.91)	0.96 (0.34–2.55)	0.85 (0.09–3.99)	4.2%
***PDGFRB***	25.23 (23.38–26.21)	1.00 (0.21–1.89)	1.02 (0.13–4.60)	3.1%
***PIK3CA***	25.92 (25.19–27.51)	1.00 (0.67–1.76)	1.14 (0.52–5.24)	3.1%
***FGFR1***	24.84 (23.62–26.29)	1.00 (0.25–1.84)	0.37 (0.08–4.48)	2.1%
***FGFR3***	25.23 (22.55–32.47)	1.00 (0.01–2.95)	0.73 (0.03–4.38)	2.1%
***IGF2***	24.46 (22.23–26.06)	1.00 (0.15–2.91)	0.32 (0.00–3.90)	2.1%
***SRC***	26.69 (24.87–30.29)	1.00 (0.12–2.11)	1.39 (0.68–3.23)	2.1%
***VEGFR3***	28.51 (26.93–29.74)	1.00 (0.48–2.35)	0.61 (0.14–3.52)	2.1%
***SMO***	27.75 (26.01–30.00)	1.00 (0.34–2.45)	0.65 (0.06–4.77)	2.1%
***DLL4***	27.87 (26.26–29.83)	1.00 (0.33–2.76)	0.98 (0.38–3.63)	2.1%
***ERBB2***	25.75 (23.78–29.99)	1.00 (0.10–2.36)	0.42 (0.08–3.56)	1.0%
***FGFR2***	25.76 (23.33–33.59)	1.00 (0.00–2.11)	0.45 (0.07–4.06)	1.0%
***KIT***	28.19 (25.45–30.88)	1.00 (0.21–2.80)	0.26 (0.05–3.44)	1.0%
***ERBB3***	25.96 (23.59–31.12)	1.00 (0.05–2.86)	0.48 (0.08–1.54)	0.0%
***ERBB4***	29.95 (26.09–34.76)	1.00 (0.03–2.97)	0.03 (0.00–1.99)	0.0%
***HGF***	29.70 (27.66–31.12)	1.00 (0.26–2.91)	0.33 (0.05–2.24)	0.0%
***CSF1R***	25.97 (24.54–27.46)	1.00 (0.47–2.38)	0.76 (0.00–2.70)	0.0%
***PDGFRA***	25.27 (24.13–25.94)	1.00 (0.35–1.99)	0.47 (0.07–2.40)	0.0%
***DDR2***	26.76 (24.66–27.60)	1.00 (0.29–2.70)	0.52 (0.07–1.75)	0.0%
***VEGFR1***	26.04 (24.26–27.96)	1.00 (0.30–2.82)	0.88 (0.25–2.68)	0.0%
***VEGFR2***	27.26 (25.74–28.42)	1.00 (0.35–1.65)	0.46 (0.12–1.78)	0.0%
***VEGFB***	26.33 (24.53–30.32)	1.00 (0.16–2.52)	0.63 (0.09–1.74)	0.0%
***STAT3***	23.32 (22.00–24.61)	1.00 (0.57–2.27)	0.84 (0.24–1.64)	0.0%
***MDM2***	24.71 (22.75–26.12)	1.00 (0.39–1.73)	0.86 (0.27–2.83)	0.0%
***ABL1***	25.22 (24.22–26.48)	1.00 (0.52–2.95)	0.69 (0.20–2.03)	0.0%
***NOTCH1***	27.82 (25.93–29.22)	1.00 (0.36–2.03)	0.71 (0.12–2.23)	0.0%
***NOTCH2***	26.13 (25.14–27.75)	1.00 (0.47–2.15)	0.72 (0.16–1.67)	0.0%
***NOTCH4***	27.72 (26.51–29.10)	1.00 (0.42–2.95)	0.65 (0.07–2.40)	0.0%
***JAK2***	25.75 (24.28–26.87)	1.00 (0.41–2.58)	0.48 (0.07–2.51)	0.0%
***TEK***	27.20 (25.85–28.30)	1.00 (0.24–2.49)	0.29 (0.07–1.42)	0.0%
***AKT1***	24.72 (23.64–26.40)	1.00 (0.66–1.61)	1.12 (0.49–2.15)	0.0%
***CCND1***	23.87 (21.70–27.36)	1.00 (0.14–2.91)	1.44 (0.19–13.85)	27.0%
***MKI67***	27.36 (25.13–35.33)	1.00 (0.00–1.58)	2.39 (0.61–13.37)	31.3%

a Median (range) of gene Ct (Cycle threshold) values.

b Median (range) of gene mRNA levels. The mRNA values of the samples were normalized such that the median of the 27 normal head and neck mRNA values was 1.

The mRNA levels of *ALK* and *ROS1* were low (Cycle Threshold, Ct > 32) in the normal head and neck tissue. Only 4 (4%) tumor samples showed *ALK* overexpression. *ROS1* was highly differentially expressed across tumor samples: mRNA levels were high (Cycle Threshold, Ct < 32) in 63% and low (Cycle Threshold, Ct > 32) in 38% of samples. Immunohistochemical analysis (IHC) of ALK and ROS1 was considered IHC-negative (H score = 0 to 1) in some of the most overexpressed samples for these 2 genes (data not shown).

We also sought for *EGFR*_VIII_ variant expression in our series of 96 HNSCCs, only 3 tumors showed a very low expression of this variant (Ct≈35) (data not shown).

### HRAS, NRAS, KRAS and PIK3CA mutations

Mutation frequencies of classical theragnostic genes *HRAS*, *NRAS* and *PIK3CA* were 3.1%, 1.0% and 8.3% respectively. No *KRAS* mutation was identified. None of the 3 *PIK3CA* mutated tumors had *PIK3CA* overexpression.

### mRNA levels of EGFR, MET and CDK6 compared to protein expression

We detected specific expression for EGFR, MET and CDK6 proteins in tumor cells of all tumor samples studied by IHC. Groups of three tumor samples studied by IHC were selected from the most RNA-overexpressed samples for *EGFR*, *MET* or *CDK6,* respectively. Three additional groups of tumor samples were selected from normally expressed samples for both *EGFR*, *MET* and *CDK6.*

**Figure 1:**
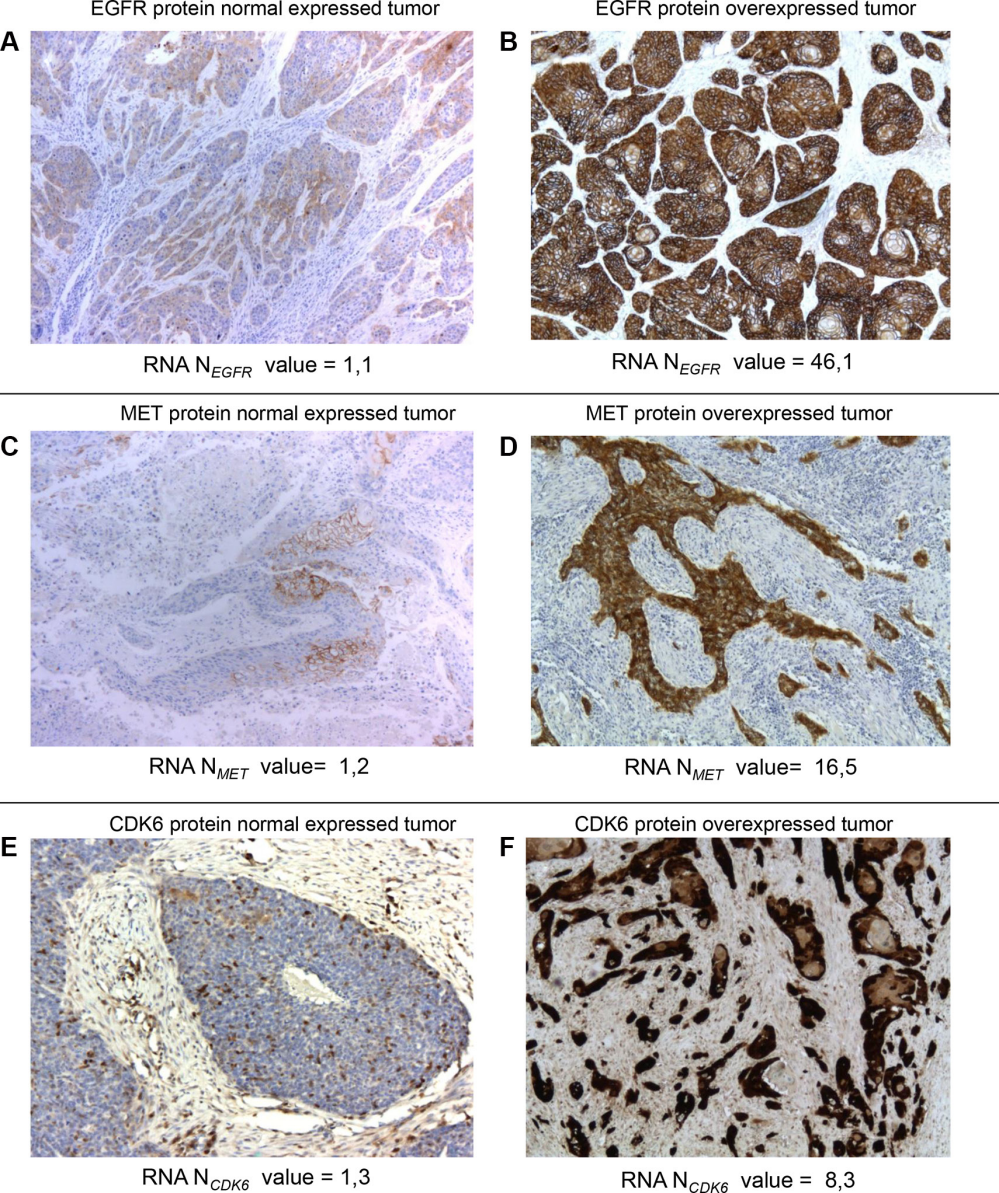
Normal and overexpressed tumors at the protein and mRNA levels for *EGFR*, *MET* and *CDK6* . Immunohistochemical staining for EGFR (**A**, **B**), MET (**C**, **D**) and CDK6 (**E**, **F**) proteins in HNSCC tumors. Examples of three tumors with *EGFR* (A), *MET* (C) and *CDK6* (E) normal mRNA-expressions and three tumors with *EGFR* (B), *MET* (D) and *CDK6* (F) mRNA-overexpressions. Intense EGFR (B), MET (D) and CDK6 (F) immunoreactivity was found in tumor epithelial cells from the *EGFR, MET, CK6* mRNA-overexpressing tumors but not in cells from the tumor without *EGFR, MET, CK6* mRNA-overexpression (A, C, E) (original magnification × 50).

A moderate to intense cytoplasmic staining of tumor cells (H score = 2 to 3) with EGFR and MET Abs, and a moderate to intense nuclear staining of tumor cells (H score = 2 to 3) with CDK6 Abs were considered to define IHC positivity (Figure 1).

We detected strong specific immunoreactivity in tumor cells of the 3 tumors which had RNA-overexpression for *EGFR*, *MET* and *CDK6*. On the contrary, none of the 3 tumors which were normally expressed for *EGFR*, *MET* and *CDK6* mRNA were considered IHC positive. We thus obtained a strong match between *EGFR*, *MET* and *CDK6* mRNA overexpression and IHC positivity (Figure 1).

### Relationship between gene expressions of the 6 most overexpressed genes

We tested, using the Spearman rank correlation test, the possible relation between mRNA levels of the 6 most overexpressed genes: *PGF, PDL1/CD274, CDK6, MET, EGFR* and *VEGFA,* as well as the proliferation marker *MKI67*. We observed a marked positive association (i.e. Spearman's rank correlation coefficient r > 0.3) between *MET* and *CDK6* (*p* < 0.001), *PGF* and *VEGFA* (*p* < 0.001), *EGFR* and *CDK6* (*p* = 0.001) (Table 3). No marked correlation could be observed with *MKI67* expression.

**Table 3:** Relationship between gene expressions of the 6 most overexpressed genes in our series of 96 HNSCCs

	CDK6	EGFR	PGF	MET	VEGFA	PDL1
***CDK6***	1 a					
	< 0.0000001					
***EGFR***	0.33	1				
	**0.0012**	< 0.0000001				
***PGF***	0.088	0.275	1			
	0.4	**0.0066**	< 0.0000001			
***MET***	0.384	0.209	0.119	1		
	**0.00016**	**0.039**	0.25	< 0.0000001		
***VEGFA***	−0.049	0.22	0.351	0.114	1	
	0.64	**0.029**	**0.00056**	0.27	< 0.0000001	
***PDL1***	0.09	0.017	0.1	−0.019	−0.083	1
	0.39	0.86	0.33	0.85	0.43	< 0.0000001
***MKI67***	0.01	0.05	−0.119	−0.091	0.256	0.034
	0.92	0.63	0.25	0.38	**0.011**	0.74

a Spearman rank correlation test.

Qualitatively, 8 of the *VEGFA* overexpressed tumors (8/13, 62%) were also *PGF* overexpressed, as compared to only 21 (21/83, 25%) of the *VEGFA* non-overexpressed tumors. We observed, using Chi2-square test, a positive association between *VEGFA* overexpression status (N_*target*_ > 3) and *PGF* overexpression (*p* = 0.02).

### Prognostic value of overexpressed genes

**Figure 2:**
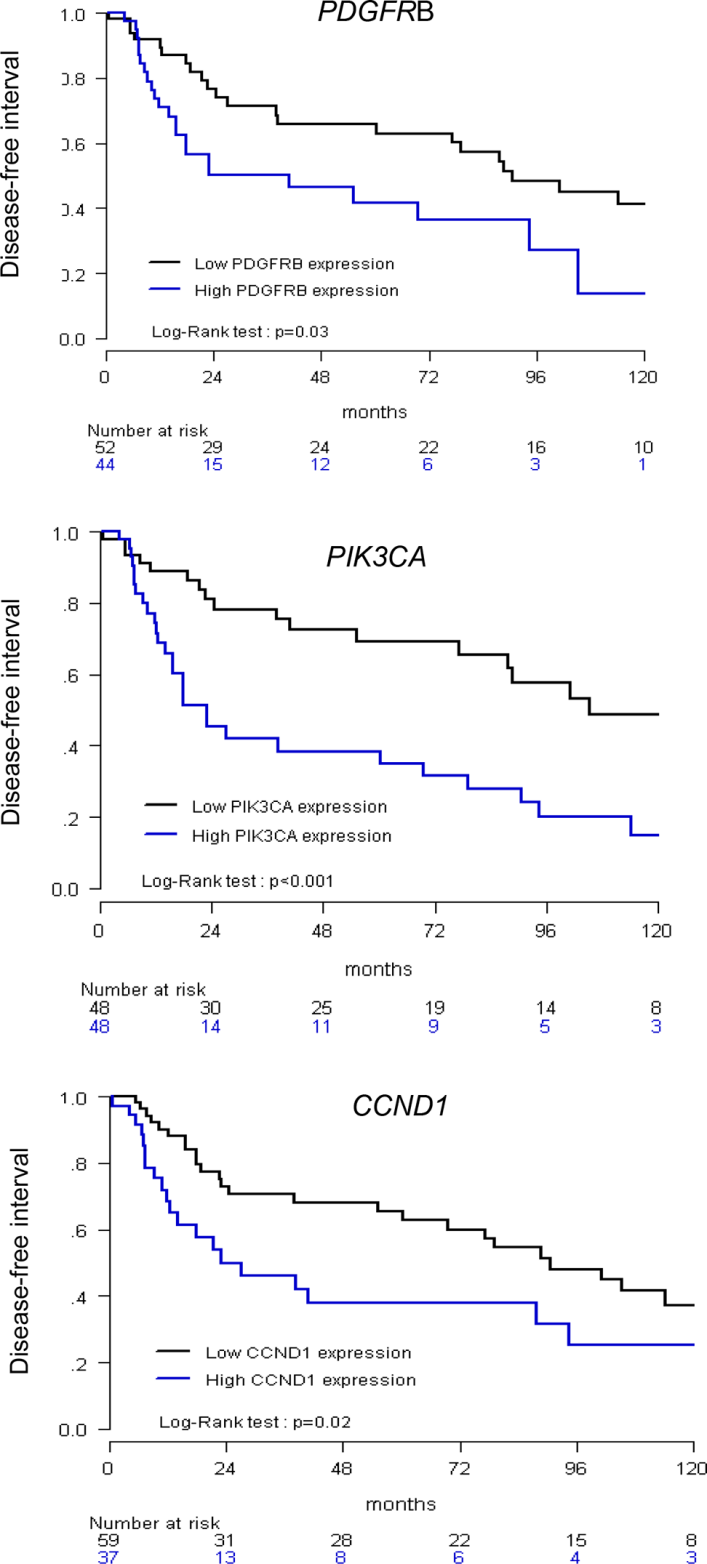
Relationship between disease-free interval (DFI) and *CCND1*, *PIK3CA* and *PDGFRB* expression High versus low expression was determined according to an optimal cut-off.

To further investigate whether mRNA expression of the 12 most frequently overexpressed genes (*PGF, PDL1/CD274, CDK6, MET, EGFR, VEGFA, IGF1R, RET, CDK4, KITLG, PDGFRB* and *PIK3CA)* could be of prognostic relevance, the log-rank test was used to identify relations between DFI (disease-free interval) and mRNA expression (Table 4). A total of 45 events has been observed. Results showed that DFI was significantly influenced by *PDGFRB* (*p* = 0.0055), *PIK3CA* (*p* = 0.03) overexpression status (N_*target*_>3), as well as *CCND1* overexpression status (*p* < 0.001), used as control. Area under curve analyses (as determined in Materials and methods section) were then performed to identify a putative cut-point by which to divide the cohort into 2 relevant gene expression subgroups for *PDGFRB*, *PIK3CA* and *CCND1*. Results confirmed that the DFI of patients with high *PDGFRB* expressing tumors was shorter than the DFI of patients with low *PDGFRB* expressing tumors (*p* = 0.03). Same results were observed for patients with high *PIK3CA* expressing tumors (*p* < 0.001), as well as for patients with high *CCND1* expressing tumors (*p* = 0.02), used as controls (Figure 2).

**Table 4:** Relationship between most overexpressed genes mRNA levels and disease-free interval (DFI) in the 96 HNSCC

Gene mRNA expression	Population (%)	Events (%)	*p*-value^a^	Gene mRNA expression according to optimal cut-off	Population (%)	Events (%)	*p*-value^a^
							
Whole population (%)	96 (100)	45 (46,9)					
							
***PGF***							
No overexpression	67 (69.8)	30 (44.8)	0.77 (NS)				
Overexpression	29 (30.2)	15 (51.7)					
***PDL1***							
No overexpression	69 (71.9)	34 (49.3)	0.36 (NS)				
Overexpression	27 (28.1)	11 (40.7)					
***CDK6***							
No overexpression	76 (79.2)	37 (48.7)	0.62 (NS)				
Overexpression	20 (20.8)	8 (40.0)					
***MET***							
No overexpression	79 (82.3)	37 (46.8)	0.28 (NS)				
Overexpression	17 (17.7)	8 (47.1)					
***EGFR***							
No overexpression	83 (86.5)	38 (45.8)	0.74 (NS)				
Overexpression	13 (13.5)	7 (53.8)					
***VEGFA***							
No overexpression	83 (86.5)	40 (48.2)	0.46 (NS)				
Overexpression	13 (13.5)	5 (38.5)					
***IGF1R***							
No overexpression	88 (91.7)	41 (46.6)	0.98 (NS)				
Overexpression	8 (8.3)	4 (50.0)					
***RET***							
No overexpression	89 (92.7)	40 (44.9)	0.35 (NS)				
Overexpression	7 (7.3)	5 (71.4)					
***CDK4***							
No overexpression	89 (92.7)	41 (46.1)	0.44 (NS)				
Overexpression	7 (7.3)	4 (57.1)					
***KITLG***							
Normal expression	92 (95.8)	43 (46.7)	0.81 (NS)				
Overexpression	4 (4.2)	2 (50.0)					
***PDGFRB***				***PDGFRB***			
No overexpression < 3	93 (96.9)	42 (45.2)	0.0055	Low expression ≤ 1.14	52 (54.1)	21 (40)	**0.03**
Overexpression > 3	3 (3.1)	3 (100)		High expression >1.14	44 (45.9)	24 (54.5)	
***PIK3CA***				***PIK3CA***			
No overexpression < 3	93 (96.9)	43 (46.2)	0.03	Low expression ≤ 1.15	50 (52.1)	18 (36)	**< 0.001**
Overexpression > 3	3 (3.1)	2 (66.7)		High expression > 1.15	46 (47.9)	27 (58.7)	
***CCND1***				***CCND1***			
No over expression < 3	70 (89.6)	8 (30.8)	< 0.001	Low expression ≤ 2	59 (61.4)	24 (40.7)	**0.02**
Overexpression > 3	26 (10.4)	18 (69.2)		High expression > 2	37 (38.6)	21 (56.7)	

a Hazard ratio.

b 95% Confidence Interval.

c Multivariate analysis.

Univariate analysis (log-rank test) thus showed that DFI was significantly linked to HPV status (*p* = 0.036), and same trend was observed for tumor location (*p* = 0.072) (Table 1). DFI was also significantly influenced by *PDGFRB, PIK3CA* and *CCND1* mRNA high expression status (*p* = 0.03, *p* < 0.001 and *p* = 0.02, respectively) (Figure 2). Multivariate analysis was performed including these 5 parameters (*p* < 0.1 in univariate analysis, Table 1): only *PIK3CA* (*p* = 0.0055) high expression remained an independent significant prognostic factor for DFI, but not *PDGFRB* (*p* = 0.10) and *CCND1* (*p* = 0.22) expression, HPV status (*p* = 0.21) and tumor location (*p* = 0.42) (Table 5).

**Table 5:** Multivariate analysis of DFI in our series of 96 HNSCCs

		HR^a^	95% CI^b^	*p*-value^c^
**RNA expression levels**				
***PDGFRB***	Low ≤ 1.14	1		
	High > 1.14	1.66	0.91–3.03	0.10 (NS)
***PIK3CA***	Low ≤ 1.15	1		
	High > 1.15	2.61	1.33–5.15	**0.0055**
***CCND1***	Low ≤ 2	1		
	High > 2	1.49	0.79–2.81	0.22 (NS)
**Clinico-pathological parameters**				
**HPV**	Negative	1		
	Positive	0.38	0.11–1.29	0.12 (NS)
**Tumor Location**	Oral cavity	1		
	Oropharynx	0.87	0.64–1.18	0.39 (NS)
	Hypopharynx	0.76	0.42–1.40	
	Larynx	0.67	0.27–1.97	

a Hazard ratio.

b 95% Confidence Interval.

c Multivariate analysis.

We also sought links between *PIK3CA* expression (low *versus* high) and standard clinicopathological and biological factors in HNSCC. Significant positive associations were observed between patients with high *PIK3CA* expressing tumors and male gender (*p* = 0.0089), alcohol consumption (*p* = 0.013), tobacco use (*p* = 0.036), high AJCC Stage (*p* = 0.014) and hypopharynx location (*p* = 0.0011) (Table S3).

## DISCUSSION

Among the 42 genes analyzed, our study reveals that 6 are relevant targets in head and neck squamous cell carcinoma: *PGF, PDL1/CD274, CDK6, EGFR, MET, VEGFA*. Almost a third of the tumors harbored an alteration of *PGF*, more than a quarter an alteration of *PDL1/CD274,* a fifth an alteration of *CDK6* and between 10 to 20% of the tumors presented *EGFR, MET* or *VEGFA* alterations. By using immunohistochemical analysis, we showed that overexpressed *EGFR*, *MET* and *CDK6* transcripts translated into overexpressed proteins, suggesting that these 3 gene expressions are mainly dysregulated at the transcriptional level in HNSCC.

All these 6 genes (*PGF, PDL1/CD274, CDK6, EGFR, MET, VEGFA*) are involved in crucial tumorogenesis pathways and thus their related proteins represent potential targets for new drugs. PGF and VEGFA are ligands of angiogenesis. CDK6 participates to the regulation of G1 restriction point, S phase entry and cell proliferation. EGFR activation plays an important role in malignant cell proliferation, angiogenesis, metastasis and inhibition of apoptosis, and PD1 plays a critical role in tumor immune evasion.

At the DNA level, we focused on 4 main oncogenes: *HRAS, NRAS, KRAS* and *PIK3CA*. Despite *RAS* family is known to be a marker of cetuximab resistance, *RAS* mutations appeared to be infrequent in our series (5%, 3 *HRAS* and 1 *NRAS* mutated tumors), and do not make *RAS* an attractive target. The incidence of PIK3CA mutations in our study is in the same range than reported in COSMIC. *PIK3CA* mutations are also known to be a marker of cetuximab resistance [8]. Interestingly, *PIK3CA* high expression was an independent unfavorable prognostic marker. This finding may have therapeutic implications. Agents that targets PI3K/AKT/mTOR pathway have already been evaluated in HNSCC. Temsirolimus demonstrated a progression-free survival rate of 40% in patients refractory to cetuximab and platinum [9]. Everolimus in combination with induction chemotherapy provided encouraging efficacy results with an overall response rate of 79% in locally advanced tumors [10]. A partial response in a heavily pretreated patient harboring a *PIK3CA* mutation was observed with BYL719, an α-specific PI3K inhibitor [11]. On the opposite, no improvement has been observed with the addition of the panPI3K inhibitor PX-866 to docetaxel in patients with advanced HNSCC without any molecular selection [12].

These discrepancies enhanced the need of predictive markers for PI3K inhibitors. It would also be worth to evaluate these agents specifically in a poor prognostic context and in an enriched population with *PIK3CA* mutations and/or overexpression. The correlation we observed between *PIK3CA* overexpression and clinicopathological and biological characteristics has previously been reported [13, 14].

Our findings are concordant with the TCGA data [4]. In both series, we observed alterations of tyrosine kinase receptors, *RAS* and *PIK3CA* pathways, cell cycle regulation and also immune evasion. However, there are important differences in terms of prevalences. We observed only 8% of *PIK3CA* mutations, whereas 21% are described in TCGA. At the RNA level we observed 3% of overexpression of *PIK3CA* versus 22% in TCGA series. These differences may be explained by the patient population with different tumor site and HPV status. In our series 45% of tumors derived from oral cavity versus 62% in TCGA. We included 17% of hypopharynx tumors *versus* none in TCGA. Only 12% of our tumors were HPV positive but a quarter was HPV positive in TCGA. These observations are crucial as we know that HPV induces a specific tumorogenesis and *PIK3CA* alterations are enriched in HPV positive population [15]. Noteworthy, tumor stage was not found to be a significant prognostic factor in our study. This might be due to the limited number of small tumors.

Our findings are noticeable because targeted drugs against the 6 relevant genes are available. In addition for 3 of these genes (*EGFR*, *MET* and *CDK6*), the good correlation between gene expression and protein expression by IHC would allow a wide and easy screening in clinical practice. To date, cetuximab is the only targeted drug used in HNSCC, but all HNSCC patients are eligible to this therapy without any molecular selection. Our data suggest that eligible tumors for cetuximab could be selected from the 13.5% *EGFR* RNA-overexpressed HNSCC. Indeed, the 13 EGFR-overexpressed samples in our series did not have any *HRAS, NRAS, KRAS* or *PIK3CA* mutation. New drugs have been developed and evaluated in other cancer types and might be interesting in HNSCC. Small inhibitor molecules of CDK 4/6 (e.g. palbociclib) have been currently approved in metastatic breast cancer by FDA and the EMA authorization is pending [16]. A clinical trial is evaluating the combination of cetuximab and palbociclib in recurrent or metastatic HNSCC (NCT02499120). MET inhibitors are also available and are currently evaluated (e.g. tivantinib, cabozantinib, crizotinib). These last years, immunotherapy has made its come-back with immune checkpoint inhibitors (e.g. pembrolizumab, nivolumab). In recurrent and/or metastatic HNSCC, Seiwert *et al.* have already reported a 50% of disease control rate (25% of overall response and 25% disease stabilization) in an expansion cohort with pembrolizumab [17]. Several phase II and III trials are currently ongoing and evaluate pembrolizumab in monotherapy or in combination with chemotherapy in recurrent and/or metastatic HNSCC (NCT02252042, NCT02358031, NCT02255097). Despite disappointing results with first generation of antiangiogenics, angiogenesis remains an important step in tumorigenesis [18, 19]. VEGF-trap is a second generation antiangiogenic agent. Like bevacizumab, it neutralizes all VEGF-A isoforms but it also inhibits other antiangiogenic ligands: VEGF-B and PGF. As, *PGF* is overexpressed in approximately 30% of HNSCC in our series, and correlates with *VEGFA* overexpression, its ability to predict response warrants further investigation.

The overexpressed genes identified in our series were only overexpressed in a fraction of HNSCC. It will therefore be necessary to test their value as predictive biomarkers of response to targeted drugs. Window of opportunity trials are good tools to assess their values. Some studies are already ongoing (NCT01415674- PREDICTOR trial and NCT01538381) which aims to identifying predictive and pharmacodynamics biomarkers of efficacy to afatinib, a dual EGFR/HER2 inhibitor. Given the high number of statistical performed in our study, we cannot exclude false positive results. Our results will have to be validated on an independent cohort of patients.

In conclusion, we identified druggable overexpressed genes associated with a poor outcome. Our data need to be confirmed in another cohort but these findings might be of interest for personalizing treatment of HNSCC patients.

## MATERIALS AND METHODS

### Patients and samples

Patients met the following criteria: primary non metastatic HNSCC for which complete clinical, histological and biological data were available; treatment with primary surgery (no radiotherapy or chemotherapy before surgery); and full follow-up at Institut Curie. All patients signed a consent form mentioning that their tumor samples might be used for scientific purposes.

Twenty seven of adjacent normal head and neck tissue from HNSCC patients were used as sources of normal RNA. Frozen tumors were used for RNA extraction. A tumor sample was considered suitable for our study if it was extracted from primary tumor with a proportion of tumor cells exceeding 70%.

### Real-time RT-PCR

The theoretical and practical aspects of real-time quantitative PCR have previously been described in detail [7].

Quantitative values were obtained from the cycle number (Cycle Threshold, Ct value) at which the increase in the fluorescence signal associated with exponential growth of PCR products started to be detected. Detection is performed by the laser detector of the ABI Prism 7900 Sequence Detection System (Perkin-Elmer Applied Biosystems, Foster City, CA), using PE Biosystems analysis software according to the manufacturer's manuals.

As the precise amount of total RNA added to each reaction mix (based on optical density) and its quality (i.e., lack of extensive degradation) are both difficult to assess, we also quantified transcripts of an endogenous RNA control gene. *TBP* (Genbank accession NM_003194) [20], which encodes the TATA box-binding protein (a component of the DNA-binding protein complex TFIID), was selected as an endogenous control because the prevalence of its transcripts is moderate, and because there are no known *TBP* retropseudogenes (retropseudogenes lead to co-amplification of contaminating genomic DNA and thus interfere with RT-PCR, despite the use of primers in separate exons).

Each sample was normalized on the basis of its *TBP* content. Results, expressed as N-fold differences in target gene expression relative to the *TBP* gene and termed “N*target*,” were determined as N*target* = 2^ΔCtsample^, where the ΔCt value of the sample is determined by subtracting the average Ct value of the target gene from the average Ct value of the *TBP* gene [7, 20]. The N*target* values of the samples were subsequently normalized such that the median of the 27 normal head and neck tissue Ntarget values was 1. RNA extraction, cDNA synthesis, and PCR conditions were as described [7].

Browsing literature [21, 22] helped us to choose a panel of 42 oncogenes coding for the major proteins directly targeted by drugs used to treat other cancers, or for proteins targeted in ongoing HNSCC clinical trials. The 42 target genes of this study are listed in Table S1. We also analyzed *MKI67* and *CCND1* mRNA levels as prognostic controls [23].

Primers for *TBP*, *CCND1, MKI67* and the 42 target genes were designed with the assistance of Oligo 6.0 computer program (National Biosciences, Plymouth, MN). We searched the dbEST and nr databases to confirm the total gene specificity of the nucleotide sequences chosen as primers and the absence of single nucleotide polymorphisms. In particular, the primer pairs were selected to be unique relative to the sequences of closely related family member genes or of the corresponding retropseudogenes. To avoid amplification of contaminating genomic DNA, one of the two primers was placed at the junction between two exons or on two different exons. Agarose gel electrophoresis was used to verify the specificity of PCR amplicons. The nucleotide sequences of the oligonucleotide primers used to amplify *CCND1, MKI67* and the 42 target genes are shown in Table S2.

### HPV genotyping

HPV status has been assessed in the Pathology Department of Institut Curie. Total DNA, isolated from formalin-fixed tissue blocks, was used for HPV typing. Real-time PCR using Sybr^®^Green and specific primers for HPV16 and 18, was performed on a 7900HT Fast Real-Time PCR System (Applied Biosystems, Foster City, CA).

### Mutations assessment

HRM primers for screening mutations were designed for *HRAS, NRAS, KRAS* (*RAS* exon 2, 3, 4), and *PIK3CA* (exon 9 and 20). PCR for HRM analysis was performed on a 384-well plate in the presence of the fluorescent DNA intercalating dye, LCgreen (Idaho Technology) in a LightCycler480^®^ (Roche). All samples were tested in duplicate. HRM analysis was performed on the Genescan software (Roche). All samples including the wild-type were plotted according to their melting profiles on the differential plot graph. All samples were sequenced using Sanger sequencing approach, as soon as an abnormal HRM curve was suspected.

### Immunohistochemistry

We performed immunohistochemical assay by using EGFR (InVitrogen, monoclonal, mouse, 31G7, 1/40, Trypsine), MET (Spring Biosciences, monoclonal, rabbit, SP44, 1/100, pH6) and CDK6 (GeneTex, monoclonal, rabbit, EPR4515, 1/100, pH 6) antibodies (Abs). IHC was performed in some samples harboring normal (N_*target*_= 1) and high expression (N_*target*_ >3) levels for *EGFR, MET* and *CDK6*.

Sections of 3 μm were cut from the paraffin-embedded tissue blocks of HNSCCs and normal head and neck tissue. Tissue sections were deparaffinized and rehydrated through a series of xylene and ethanol washes. All immunostaining was processed by using a LEICA (BOND III) automated immunostaining device. The specificity of the antibodies was confirmed by doing immunohistochemical studies with the same protocol on paraffin-embedded human tissue sections containing lymphocytes. A semi-quantitative histological score (H score = intensity × frequency) was performed (score 0 = negative staining, score 1 = weak staining, score 2 = moderate staining, score 3 = strong staining).

### Statistical analysis

The distributions of target mRNA levels were characterized by their median values and ranges. Relationships between mRNA levels of the different target genes, and clinical, biological and pathological parameters, were identified by using parametric test, namely the Chi-square test, and non-parametric tests such as Kruskal-Wallis test, and the Spearman rank correlation test. Differences between 2 populations were considered significant at confidence levels greater than 95% (*P* < 0.05).

Disease-free interval (DFI) was determined from the time of initial diagnosis to the time of the first event among local recurrence, metastatic recurrence or second cancer. Survival distributions were estimated by the Kaplan-Meier method, and the significance of differences between survival rates was ascertained with the log-rank test. Univariate and multivariate Cox proportional hazard models were performed to identify the clinical and molecular markers that impact the DFI. The results are presented as hazard ratios and 95% confidence intervals (CIs). To visualize the efficacy of a molecular marker (gene expression level) to discriminate two populations (patients that remained/or not disease-free at latest follow-up) in the absence of an arbitrary cut-off value, data were summarized in a ROC (receiver operating characteristic) curve [24]. The AUC (area under curve) was calculated as a single measure for discriminate efficacy. A *P* value less than 0.05 was considered to be statistically significant, except for univariate analysis that considered a *P* value less than 0.10.

## SUPPLEMENTARY MATERIALS TABLES



## Abbreviations

AJCC: American Joint Committee on Cancer
AUC: Area Under Curve
DFI: Disease Free Interval
EGFR: Epidermal Growth Factor Receptor
HNSCC: Head and neck squamous cell carcinoma
HPV: Human Papilloma Virus
HRM: High Resolution Melting
IHC: Immunohistochemistry
PCR: Polymerase Chain Reaction
ROC: Receiver Operating Characteristic
RT-PCR: Reverse transcription Polymerase Chain Reaction
TCGA: The Cancer Genome Atlas
