# Supplementary material for: Identification of new candidate therapeutic target genes in head and neck squamous cell carcinomas
Source: Oncotarget. 2016 Jun 18;7(30):47418–30. doi: 10.18632/oncotarget.10163 (PMC5216951; doi:10.18632/oncotarget.10163)
Supplement: Supplementary file 1 [file oncotarget-07-47418-s001.pdf]

Identification of new candidate therapeutic target genes in head and neck squamous cell carcinomas

Supplementary Materials

Supplementary Table S1: Selection of targeted therapies matching 42 selected druggable genes

Signalling pathway	Gene symbols	Names of genes	Chromosome location	Genbank accession number	Selected therapies
**Growth factors, tyrosine kinase receptors, and ligands**	***EGFR***	epidermal growth factor receptor	7p12	NM_005228.3	cetuximab, gefitinib, erlotinib, lapatinib, afatinib, neratinib, panitumumab
	ERBB2	erb-b2 receptor tyrosine kinase 2	17q12	NM_004448.3	trastuzumab, lapatinib, afatinib, neratinib
	ERBB3	erb-b2 receptor tyrosine kinase 3	12q13	NM_001982.3	pertuzumab, MM121
	ERBB4	erb-b2 receptor tyrosine kinase 4	2q33.3-q34	NM_005235.2	neratinib
	FGFR1	fibroblast growth factor receptor 1	8p11.23-p11.22	NM_015850.3	vandetanib, PD173074
	FGFR2	fibroblast growth factor receptor 2	10q26	NM_000141.4	vandetanib, PD173075
	FGFR3	fibroblast growth factor receptor 3	4p16.3	NM_000142.4	vandetanib, PD173076, dovitinib
	IGF1R	insulin-like growth factor 1 receptor	15q26.3	NM_000875.4	linsitinib, dalotuzumab
	IGF2	insulin-like growth factor 2	11p15.5	NM_000612.5	
	MET	MET proto-oncogene, receptor tyrosine kinase	7q31	NM_000245.2	crizotinib, foretinib
	HGF	hepatocyte growth factor (hepapoietin A, scatter factor)	7q21.1	NM_000601.4	
	ALK	anaplastic lymphoma receptor tyrosine kinase	2p23	NM_004304.4	crizotinib
	ROS1	ROS proto-oncogene 1 , receptor tyrosine kinase	6q22	NM_002944.2	
	PDGFRA	platelet-derived growth factor receptor, alpha polypeptide	4q12	NM_006206.4	imatinib, sunitinib, sorafenib, pazopanib, vatalanib, tandutinib, nilotinib, motesanib, vandetanib
	PDGFRB	platelet-derived growth factor receptor, beta polypeptide	5q33.1	NM_002609.3	
	VEGFA	vascular endothelial growth factor A	6p12	NM_003376.5	bevacizumab
	VEGFB	vascular endothelial growth factor B	11q13	NM_003377.4	aflibercept
	PGF	placental growth factor	14q24.3	NM_002632.5	aflibercept
	VEGFR1	fms-related tyrosine kinase 1	13q12	NM_002019.4	sunitinib, sorafenib, pazopanib, vatalanib, apatinib, motesonib, cediranib, vandetanib
	VEGFR2	kinase insert domain receptor	4q11-q12	NM_002253.2	sunitinib, sorafenib, pazopanib, vatalanib, apatinib, brivanib, motesonib, cediranib, vandetanib, foretinib
	VEGFR3	fms-related tyrosine kinase 4	5q35.3	NM_002020.4	sunitinib, sorafenib, pazopanib, vatalanib, apatinib, motesonib, cediranib, vandetanib
	TEK	TEK tyrosine kinase, endothelial	9p21	NM_000459.4	bevacizumab
	KIT	v-kit Hardy-Zuckerman 4 feline sarcoma viral oncogene homolog	4q12	NM_021099.3	imatinib, sunitinib, sorafenib, pazopanib, vatalanib, tandutinib, nilotinib, apatinib, motesanib
	KITLG	KIT ligand	12q22	NM_000899.4	
	DDR2	discoidin domain receptor tyrosine kinase 2	1q23.3	NM_006182.2	imatinib, nilotinib,bafetinib
	ABL1	ABL proto-oncogene 1, non-receptor tyrosine kinase	9q34.1	NM_005157.5	imatinib, nilotinib, dasatinib

	CSF1R	colony stimulating factor 1 receptor	5q32	NM_005211.3	sunitinib, vatalanib
	SRC	SRC proto-oncogene, non-receptor tyrosine kinase	20q12-q13	NM_005417.4	dasatinib, bosutinib, apatinib
	RET	ret proto-oncogene	10q11.2	NM_020630.4	sunitinib, motesanib
**JAK-STAT pathway**	***JAK2***	janus kinase 2	9p24	NM_004972.3	lestaurtinib
	STAT3	signal transducer and activator of transcription 3 (acute-phase response factor)	17q21.31	NM_003150.3	atiprimod
**Cell Differenciation**	***NOTCH1***	notch 1	9q34.3	NM_017617.3	MK0752, PF-03084014, RO4929097, R4733
	NOTCH2	notch 2	1p13-p11	NM_024408.3	MK0752, PF-03084014, RO4929097, R4733, tarextumab
	NOTCH4	notch4	6p21.3	NM_004557.3	MK0752, PF-03084014, RO4929097, R4733
	DLL4	delta-like 4 (Drosophila)	15q14	NM_019074.3	REGN421
	SMO	smoothened, frizzled class receptor	7q32.3	NM_005631.4	vismodegib, patidegib, erismodegib, taladegib
**PI3K-AKT-mTOR**	***AKT1***	v-akt murine thymoma viral oncogene homolog 1	14q32.32	NM_005163.2	AZD5363, MK2206, perifosine, ipatasertib (GDC-0068)
	PIK3CA	phosphatidylinositol-4,5-bisphosphate 3-kinase, catalytic subunit alpha	3q26.3	NM_006218.2	buparlisib (BKM120), copanlisib, taselisib (GDC-0032)
**Cell Cycle**	***CDK4***	cyclin-dependent kinase 4	12q14.3-q15	NM_000075.3	palbociclib, RGB-286638 , R547 , P1446-05, BAY1000394, ZK304709
	CDK6	cyclin-dependent kinase 6	7q21-q22	NM_001259.6	palbociclib, R547
	MDM2	MDM2 proto-oncogene, E3 ubiquitin protein ligase	12q14.3-q15	NM_002392.5	idasanutlin, RO5503781
**Immune response**	***PDL1/ CD274***	CD274 molecule	9p24	NM_014143.3	avelumab, tremelimumab,atezolizumab, BMS-936559
**Controls**	***CCND1***	cyclin D1	11q13	NM_053056.2	_
	MKI67	marker of proliferation Ki-67	10q26.2	NM_002417.4	_

Supplementary Table S2: Primers used for quantitative RT-PCR for the 42 selected genes and *CCND1, MKI67, TBP* controls

Gene	Upper sequence (5′ à 3′)	Lower sequence (5′ à 3′)	Amplicon size (pb)
EGFR	5′ GGA GAA CTG CCA GAA ACT GAC C 3′	5′ GCC TGC AGC ACA CTG GTT G 3′	106
ERBB2	5′ AGC CGC GAG CAC CCA AGT 3′	5′ TTG GTG GGC AGG TAG GTG AGT T 3′	147
ERBB3	5′ GTCTGTGTGACCCACTGCAACT 3′	5′ GGGTGGCAGGAGAAGCATT 3′	80
ERBB4	5′ GGC TGC TGA GTT TTC AAG GAT G 3′	5′ GCT TCA TAC GAT CAT CAC CCT GA 3′	74
FGFR1	5′ GAA TTG GAG GCT ACA AGG TCC GTT 3′	5′ GGT TGA TGC TGC CGT ACT CAT TCT 3′	118
FGFR2	5′ GGC CGT GAA GAT GTT GAA AGA TG 3′	5′ CCT GTG TGC AGG CTC CAA GAA 3′	128
FGFR3	5′ GGCTGAAGAACGGCAGGGAGT 3′	5′ CTGCTGATGCCGCAGCTTGA 3′	68
IGF1R	5′ CCAAGGCCTGAAAACTCCATCT 3′	5′ ACACATTCTCGCTGATCCTCAACT3′	116
IGF2	5′ CGA CCG TGC TTC CGG ACA AC 3′	5′ AGG CGC TGG GTG GAC TGC TT 3′	85
MET	5′ ATG GGT CAA TTC AGC GAA GTC C 3′	5′ GAT CGA GAA ACC ACA ACC TGC AT 3′	119
HGF	5′ CCC TGG TGT TTC ACA AGC AAT C 3′	5′ GGT CAT GCA TTC AAC TTC TGA ACA 3′	81
KIT	5′ TCC TCG CCT CCA AGA ATT GTA T 3′	5′ CTT GAT GTC TCT GGC TAG ACC AAA 3′	111
KITLG	5′ ACG TCA CTA AAT TGG TGG CAA ATC 3′	5′ GCT TAT CCA ACA ATG ACT TGG CA 3′	98
RET	5′ GCC ACC GAC CAG CAG ACC T 3′	5′ GCC TCC TCG GCC ACA TAT GA 3′	80
CSF1R	5′ GATGGGTGGCAGGAAGGTGA 3′	5′ GGGCCCTGGGATGACTTTCT 3′	67
PDGFRA	5′ CAT TTA CAT CTA TGT GCC AGA CCC A 3′	5′ ATG GCA GAA TCA TCA TCC TCC AC 3′	93
PDGFRB	5′ CCC CAG TGC CGA GTT AGA AGA C 3′	5′ GCA CGT AGC CGC TCT CAA CC 3′	116
ALK	5′ CGG AGG ATA TAT AGG CGG CAA T 3′	5′ ATG CCC AGT GGA CTG ATG AAG GA 3′	90
SRC	5′ ACC ACC TTT GTG GCC CTC TAT G 3′	5′ GCC ACC AGT CTC CCT CTG TGT T 3′	109
DDR2	5′ TCC AGT CAC CGA CCA CTC CAT 3′	5′ GTA AGA CAC CAA GCC ATC TAG CCA 3′	82
ROS1	5′ GATGGCAACGTTTTATGGTCCTT 3′	5′ TCTAGCTGCCAGATCCCTGTGA 3′	126
VEGFA	5′ CTT GCC TTG CTG CTC TAC CTC C 3′	5′ CAT CCA TGA ACT TCA CCA CTT CGT 3′	103
VEGFB	5′ GGT GCC CAG CTG CGT GAC T 3′	5′ CCG GAT CAT GAG GAT CTG CAT C 3′	112
VEGFR1	5′ ATC ATT CCG AAG CAA GGT GTG AC 3′	5′ TCC TTC TAT TAT TGC CAT GCG CT 3′	122
VEGFR2	5′ TGG GAA CCG GAA CCT CAC TAT C 3′	5′ GTC TTT TCC TGG GCA CCT TCT ATT 3′	132
VEGFR3	5′ GTC ACG CTG CGC TCG CAA A 3′	5′ GTG CAG CAG TGG CGT GGA CA 5′	99
PGF	5′ GCTCGTCAGAGGTGGAAGTGGT 3′	5′ CTCGCTGGGGTACTCGGACA 3′	103
STAT3	5′ GCT TCC TGC AAG AGT CGA ATG TT 3′	5′ GGC TTC TCA AGA TAC CTG CTC TGA 3′	85
MDM2	5′ GCG TGC CAA GCT TCT CTG TGA 3′	5′ CTG AGT CCG ATG ATT CCT GCT GA 3′	96
ABL1	5′ GCCAGTACGGGGAGGTGTACGA 3′	5′ CTCCATGGTGTCCTCCTTCAAGGT 3′	96
NOTCH1	5′ TGC GTG CAG CGC GTC AAT GAC T 3′	5′ CAG CGG CGC CCG GTG TGA 3′	62
NOTCH2	5′ CAC CTG CCC ACA AGG CTA CA 3′	5′ CAC AAG GAT TGC TAT TGG CCA T 3′	80
NOTCH4	5′ GTGCATCTGCCTGCCTGGATT 3′	5′ CCCACCATTGGCACAGGGA 3′	85
JAK2	5′ GACTTTTGCTGTCGAGCGAGAA 3′	5′ CTTTGTCCCACTGAGGTTGTACTCTT 3′	94
SMO	5′ GCT ACT TCC TCA TCC GAG GAG TCA 3′	5′ GGC GCA GCA TGG TCT CGT T 3′	108
TEK	5′ GTC AGC TTG CTC CTT CTG GAA CT 3′	5′ GAG GCA ATG CAG GTG AGA GAT GT 3′	107
AKT1	5′ CCC AGG TCA CGT CGG AGA CT 3′	5′ ACT CCA TGC TGT CAT CTT GGT CA 3′	99
PIK3CA	5′CCTGATCTTCCTCGTGCTGCTC 3′	5′ ATGCCAATGGACAGTGTTCCTCTT 3′	92
CDK4	5′ CCA ATG TTG TCC GGC TGA TG 3′	5′ CTA CAT GCT CAA ACA CCA GGG TTA C 3′	84
CDK6	5′ CCC AAC GTG GTC AGG TTG TTT 3′	5′ GTG GTC AAG TCT TGA TCG ACA TGT T 3′	101
PDL1/ CD274	5′ GCTGAATTGGTCATCCCAGAACTAC 3′	5′ AAACGGAAGATGAATGTCAGTGCTAC 3′	95
DLL4	5′ AAT GGA GGC AGC TGT AAG GAC C 3′	5′ AAG GTG CTG TGT TCA CAA TGC AG 3′	90
MKI67	5′ ATT GAA CCT GCG GAA GAG CTG A 3′	5′ GGA GCG CAG GGA TAT TCC CTT A 3′	105

CCND1	5′ GGATGCTGGAGGTCTGCGA 3′	5′ AGAGGCCACGAACATGCAAG 3′	128
TBP	5′ TGCACAGGAGCCAAGAGTGAA 3′	5′ CACATCACAGCTCCCCACCA 3′	112

Supplementary Table S3: Association between clinical and biological characteristics of the 46 HNSCC patients with high expression levels of *PIK3CA*, compared to the patients with low expression levels of *PIK3CA*

	**Whole cohort**	***PIK3CA* high expression**	p^a
	**Number of patients (%)**	**Number of patients (%)**	
**Total**	96	46 (47.9)	
Age			
< 56	46 (53.1)	24 (52.2)	0.42
> 56	50 (46.9)	22 (44)	
Sexe			**0.0089**
male	77 (80.2)	42 (54.5)	
female	19 (19.8)	4 (21.1)	
HPV status			
positive	12 (12.5)	3 (25.0)	0.089
negative	84 (87.5)	43 (51.1)	
Alcool[b]			
Yes	50 (70.4)	28 (56.0)	**0.013**
No	21 (29.6)	5 (23.8)	
Tobacco[c]			
Yes	58 (72.5)	31 (53.4)	**0.036**
No	22 (27.5)	6 (27.3)	
AJCC Stage			
stage I	10 (10.4)	2 (20)	**0.014**
stage II	15 (15.6)	3 (20)	
stage III	12 (12.5)	7 (58.3)	
stage IV	59 (61.5)	34 (57.6)	
Tumor Location			
Oral cavity	43 (44.8)	13 (30.2)	**0.0011**
Oropharynx	20 (20.8)	9 (45)	
Larynx	17 (17.7)	10 (58.8)	
Hypopharynx	16 (16.7)	14 (87.5)	
HRAS, NRAS, PIK3CA mutations	12 (12.5)	5 (41.6)	0.64
HRAS, NRAS, PIK3CA wild type	84 (87.5)	41 (48.8)	

[a]Chi2 test.
[b]Tobacco use was considered at 10 packyears or more. Information available for 71 patients.
[c]Alcohol use was considered at 10 gr/day or more (ie. alcohol unit). Information available for 80 patients.
